# Ion Irradiation-Induced Microstructural Evolution of Ni–Mo–Cr Low Alloy Steels

**DOI:** 10.3390/ma11112268

**Published:** 2018-11-13

**Authors:** Hongying Sun, Penghui Lei, Guang Ran, Hui Wang, Jiyun Zheng, Yiyong Zhang, Zhigang Wang, Shui Qiu

**Affiliations:** 1 School of Mechanical Engineering, Anyang Institute of Technology, Anyang 455002, China; shysun029@163.com (H.S.); 20160298@ayit.edu.cn (Z.W.); 2 College of Energy, Xiamen University, Xiamen 361102, China; p.h.lei@foxmail.com; 3 Science and Technology on Reactor Fuel and Materials Laboratory, Nuclear Power Institute of China, Chengdu 610041, China; zhengjiyun@aliyun.com (J.Z.); ustbzhangyiyong@163.com (Y.Z.); qsnpic@163.com (S.Q.)

**Keywords:** ion beam irradiation, point defects, dislocations, positron annihilation

## Abstract

As leading candidates of sheet steels for advanced nuclear reactors, three types of Ni–Mo–Cr high-strength low alloy (HSLA) steels named as CNST1, CNST2 and CNSS3 were irradiated by 400 keV Fe^+^ with peak fluence to 1.4 × 10^14^, 3.5 × 10^14^ and 7.0 × 10^14^ ions/cm^2^, respectively. The distribution and morphology of the defects induced by the sample preparation method and Fe^+^ irradiation dose were investigated by transmission electron microscopy (TEM) and positron-annihilation spectroscopy (PAS). TEM samples were prepared with two methods, i.e., a focused ion beam (FIB) technique and the electroplating and twin-jet electropolishing (ETE) method. Point defects and dislocation loops were observed in CNST1, CNST2 and CNSS3 samples prepared via FIB. On the other hand, samples prepared via the ETE method revealed that a smaller number of defects was observed in CNST1, CNST2 and almost no defects were observed in CNST3. It is indicated that artifact defects could be introduced by FIB preparation. The PAS *S*-*W* plots showed that the existence of two types of defects after ion implantation included small-scale defects such as vacancies, vacancy clusters, dislocation loops and large-sized defects. The *S* parameter of irradiated steels showed a clear saturation in PAS response with increasing Fe^+^ dose. At the same irradiation dose, higher values of the S-parameter were achieved in CNST1 and CNST2 samples when compared to that in CNSS3 samples. The mechanism and evolution behavior of irradiation-induced defects were analyzed and discussed.

## 1. Introduction

Nuclear grade high-strength low alloy (HSLA) steels have been developed for a long time and the Mn–Mo–Ni low alloy steels, such as SA508 Grade 3 and SA533 Grade B, have been widely used in nuclear reactor pressure vessels (RPV) construction for more than 30 years [1], which are due to the advantage of a combination of good strength, toughness and weldability in addition to economic concerns [2,3,4,5,6,7,8,9]. With the increasing output power from a single plant, new RPV concepts, such as volume enlargement and RPV integration, have been developed. Thus, nuclear grade Ni–Mo–Cr HSLA steel plates with higher strength and toughness, i.e., SA543 and SA542 steels, have been considered as the candidates, and achieved by optimized heat treatment and/or chemistry modification such as the increase of Ni, V and Cr content. Among them, the SA543 steel was carefully investigated and found to have excellent chemical corrosion resistance, which leads to its wide applications in industrial fields, including the fabrication of high-temperature reactors and chemical containers [10]. However, there are few reports about its applications in RPV production though it had been specified in ASME Code Sect. II, let alone irradiation behavior.

Over 40 years of operation, the dose reaches ~0.1 dpa for the PWR RPV. The neutron doses in BWRs are almost one or two orders lower than those in PWRs since BWRs have lower core power densities and larger distances between the core and components than PWRs [11]. Irradiation-induced defects in the materials, i.e., dislocation loops and vacancies, and their evolution are responsible for material hardening and embrittlement behavior, which is considered to be a critical problem for the life assessment of nuclear grade steels [12,13]. Therefore, the analysis and characterization of irradiation-induced defects are of great importance to better understand the underlying mechanism of material property degradation [14].

Since the positrons are sensitive to the irradiation-induced defects such as vacancies, vacancy clusters, dislocations, etc. [15,16], the positron-annihilation spectroscopy (PAS) technique is usually used to detect tiny defects that are difficultly observed by high-resolution transmission electron microscopy (HRTEM) [9,17,18]. PAS has become a powerful tool to reveal detailed information about the irradiation-induced damage at a low damage level (<1 dpa, displacement per atom). Additionally, defect structures formed after ion implantation were investigated by PAS method [16].

In the present work, transmission electron microscopy (TEM) observations and PAS analysis were conducted to investigate the irradiation-induced defects in the nuclear grade Ni–Mo–Cr steels after Fe^+^ irradiation. Two methods of the preparation of TEM samples were conducted, i.e., focused ion beam (FIB) technique and the electroplating and twin-jet electropolishing (ETE) method. The mechanism and evolution behavior of irradiation-induced defects were analyzed and discussed.

## 2. Experiments

In this study, three newly developed nuclear grade Ni–Mo–Cr HSLA steels were used as target materials. All three, RPVs termed as CNST1, CNST2 and CNSS3, respectively, were essentially qualified with the ASME Standard SA543 and considered as candidates for the RPVs. The chemical composition of the three alloys was listed in Table 1, which was supported by the manufacturer.

The size of the samples used for metallographic analysis and ion irradiation was 10 mm × 10 mm × 3 mm. Raw materials were firstly cut from the as-received steel plates by a precision diamond knife cutting machine, then grinded with SiC sandpapers from 180 to 5000 grid, finally polished with 3~0.05 μm diamond suspensions. Before metallographic observation, the polished samples were etched chemically using a 10% HClO_4_ ethanol solution. For ion irradiation experiment, the samples were electrochemically polished using a 5% HClO_4_ ethanol solution, removing the mini scratches on the sample surface. TEM samples were first sliced vertical to the sample surface from as-received steels, then grounded by SiC sandpapers and polished using 1 μm diamond paste. Finally, the samples were twin-jet electro-polished using a 10% HClO_4_ ethanol solution to perforate for TEM observation.

**Figure 1:**
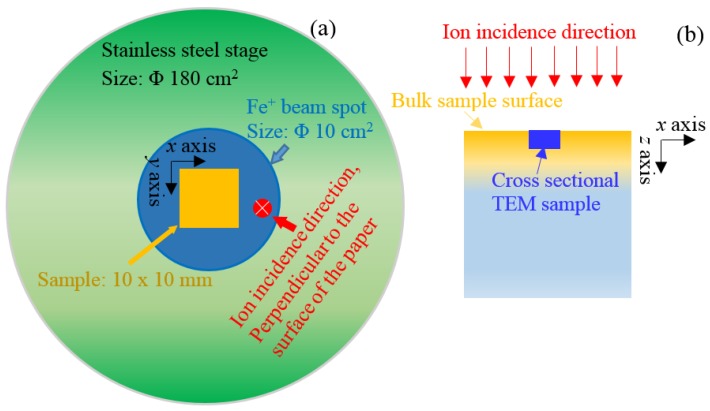
(**a**) The sketch of the samples in the ion implanter and (**b**) the location of TEM foil from the irradiated sample.

A designed 400 keV Fe^+^ ion implantation was performed at room temperature using a NEC 400 kV ion implanter in the College of Energy, Xiamen University. The sketch of the samples in the ion implanter is shown in Figure 1. The samples of 10 × 10 × 3 mm in dimensions were pasted on the surface of stainless-steel stage with Φ180 cm^2^ by carbon paste. The beam spot size of iron ions was approximately Φ10 cm^2^. The polished surface was used as ion irradiation surface. The direction of ion incidence was parallel to the normal direction of polished surface. 

**Figure 2:**
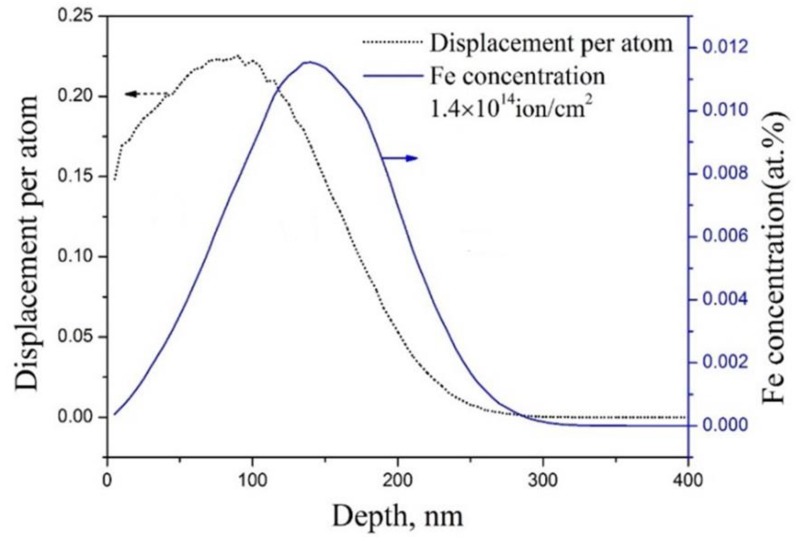
Depth profile of displacement damage and Fe^+^ concentration in the steels irradiated by 400 keV Fe^+^ with a fluence of 1.4 *×* 10^14^ ions/cm^2^ calculated by SRIM 2008 software (quick mode).

Figure 2 shows the depth distribution of displacement damage, which was simulated by the Stopping and Range of Ions in Matter (SRIM)-2008 software with quick mode [19]. The displacement energy of Fe atom was selected as 40 eV [20]. After Fe^+^ implantation, total fluence of 1.4 × 10^14^ Fe^+^/cm^2^, 3.5 × 10^14^ Fe^+^/cm^2^ and 7.0 × 10^14^ Fe^+^/cm^2^ leads to the accumulated irradiation damage of 0.2 dpa, 0.5 dpa and 1.0 dpa, respectively. The ion flux was approximately 1 × 10^11^ Fe^+^/cm^2^·s in the present work. The peak of irradiation damage has a depth of approximately 80 nm.

The cross-sectional TEM samples were prepared by two different methods and the location of TEM foil from the irradiated sample was shown in Figure 1b. Some of cross-sectional samples were prepared by FIB technique. Since high energy ions were used (normally 30 keV Ga^+^ ions) to bombard and mill TEM foils, it is believed that extra defects and amorphous structure were induced on the surface of the foils as reported in [21]. In the present work, the extra defects induced by the focused Ga^+^ ions were noticed and marked. As a comparison, the electroplating and twin-jet electropolishing method was also utilized to prepare TEM samples. The electroplating process was described by Seitzman et al. [22]. A nickel coating with approximately 2 mm thickness was deposited on the irradiated sample surface. Raw slices were first cut along the ion incident direction, then grounded to approximately 50 μm thickness, and finally punched with a Φ3 mm hole to form a TEM sample that cover Ni coating and irradiated region. The samples were first pitted by a dimple grinder and then twin-jet electropolished by a 10% HClO_4_ ethanol solution to perforation for TEM observation.

PAS measurements were conducted at room temperature using a slow-positron beam in the Key Laboratory of Nuclear Analysis Techniques, Institute of High Energy Physics in Beijing. Variable-energy positrons ranged from 0.18 to 20.18 keV were produced and annihilated, so that yielded 511 keV gamma-ray photons were detected by high-purity Ge detector. Doppler broadening spectroscopy (DBS) were recorded and accumulated up to two million counts in the tests. Obtained DBS were characterized by *S* and *W* parameters, where *S* was the ratio of the counts in the central energy region (510.24–511.76 keV in this paper), and *W* was the ratio of the wing area of the annihilation peak (513.6–517.8 keV and 504.2–508.4 keV in this paper). Additionally, *S* and *W* yielded the information of low-momentum and high-momentum electrons, respectively.

## 3. Results and Discussion

### 3.1. Microstructure

**Figure 3:**
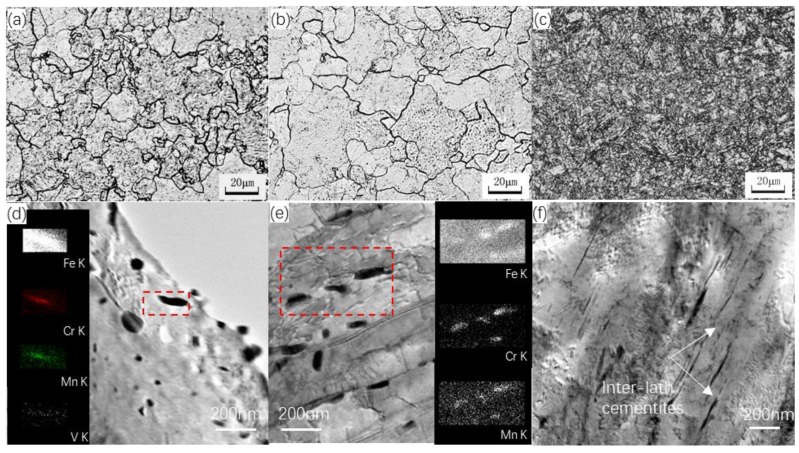
Microstructure of the as-received CNST1, CNST2 and CNSS3, (**a**–**c**) metallographs; (**d**–**f**) bright field TEM images; (**a**,**d**) CNST1; (**b**,**e**) CNST2; (**c**,**f**) CNSS3.

The metallographs and TEM images of the as-received CNST1, CNST2 and CNSS3 are shown in Figure 3. It can be seen that CNST1 and CNST2 samples present a typical ferrite in the optical observation as shown in Figure 3a,b, and a considerable number of carbide precipitates distributed in the matrix according to the bright field TEM images as shown in Figure 3d,e. Additionally, the precipitates along the grain boundaries were examined by energy dispersive spectroscopy (EDS), demonstrating their consistence of V, Cr and Mn elements. In CNST3, a mixture of tempered sorbite with a small amount of lower bainite can be observed, as well as the cementite along sub-microstructural laths as shown in Figure 3c,f.

**Figure 4:**
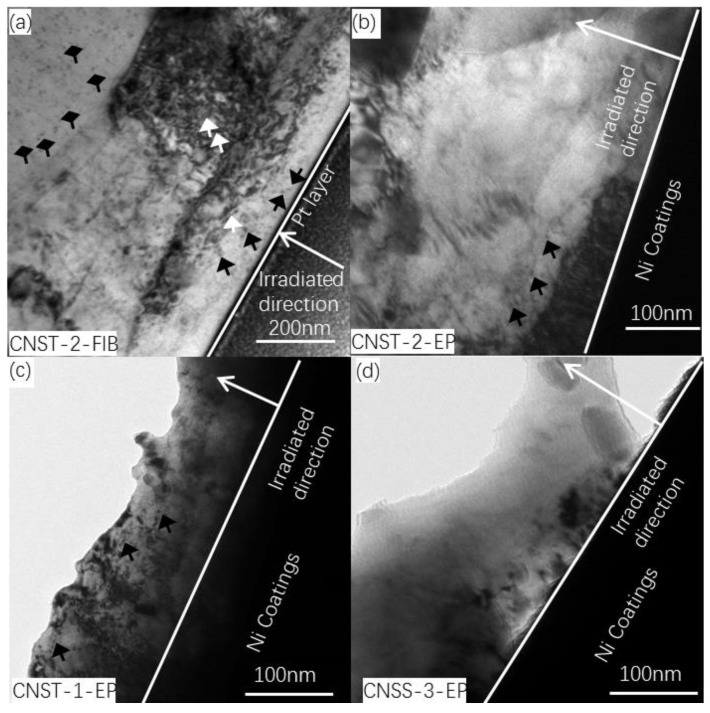
Bright field TEM images showing the microstructure of the cross-sectional samples irradiated with a fluence of 7.0 × 10^14^ Fe^+^/cm^2^, (**a**) CNST2 sample prepared with FIB; (**b**–**d**) CNST2, CNST1 and CNSS3 samples prepared by ETE method.

Bright field TEM images of the cross-sectional microstructure of these three types of steels irradiated with a fluence of 7.0 × 10^14^ Fe^+^/cm^2^ at room temperature are shown in Figure 4. Point defects and dislocation loops are observed in the steel matrix. The uniform-distribution black dots in the ferrite matrix are considered to be the point defects marked by the black arrows as shown in Figure 4a, while the small bean-shape features marked by white arrows are believed to be dislocation loops. The microstructure of CNST2 steel prepared by FIB is shown in Figure 4a. Point defects with radius approximately 5 nm and small dislocation loops around 50 nm in the irradiated area are marked by black and white arrows, respectively. A similar CNST-2 sample prepared by ETE method is shown in Figure 4b. Obviously, there are only a few irradiation-induced defects in the 100-nm depth region. None of dislocation loops can be observed in the steel matrix. Figure 4c,d present the cross-sectional microstructure of the irradiated CNST1 and CNSS3 steels prepared by ETE method, respectively. CNST1 behaved similarly as CNST2 while neither point defects nor dislocation loops are visible in CNSS3 sample. Afterwards, it is indicated that post-irradiation with fluence of 1.4 × 10^14^ Fe^+^/cm^2^ and 3.5 × 10^14^ Fe^+^/cm^2^, the size and number density of point defects are less and smaller in the samples prepared by ETE method. Also, there are no dislocation loops observed. Therefore, the dislocation loops and high number density of point defects in Figure 4a are introduced by FIB as a result of energetic Ga^+^ ions bombarded with samples. This is also regarded as the major drawback of FIB machining for the preparation of TEM samples [21]. Therefore, the ETE method gives a better way to prepare TEM samples, which are damage free during the sample preparation.

### 3.2. Irradiation-Induced Defects

**Figure 5:**
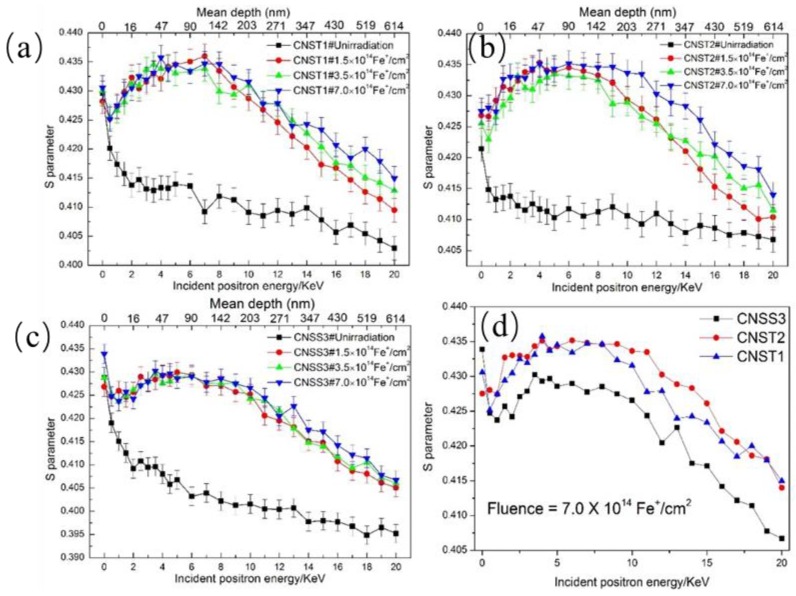
The relationship between S parameter and the positron energy, (**a**) CNST1 steel, (**b**) CNST2 steel and (**c**) CNSS3 steel, (**d**) These three types of steels irradiated with a fluence of 7.0 × 10^14^ Fe^+^/cm^2^.

Figure 5 shows the relationship between the *S* parameter of these three types of candidate steels irradiated with fluence of 1.4 × 10^14^ Fe^+^/cm^2^, 3.5 × 10^14^ Fe^+^/cm^2^ and 7.0 × 10^14^ Fe^+^/cm^2^ at room temperature and incident positron energy (mean depth of the annihilating positrons). As a comparison, the results of the pre-irradiated samples are also included.

According to the SRIM simulation results as shown in Figure 2, the peaks of irradiation damage and injected iron ion concentration are located in the depth of approximately 80 nm and 160 nm, respectively, which is consistent with the peak region of *S* parameters as shown in Figure 4. The total depth of irradiation damage and injected iron ion concentration are 300 nm and 330 nm, respectively, and are less than that of *S* parameters in irradiated sample. The reason should be due to that the crystal properties such as crystal orientation, crystal boundary, etc., phase characteristics and intrinsic defects are not considered in the SRIM software. For example, the crystal boundary is a good channel for point defect migration. Therefore, the actual irradiation depth is larger than that of SRIM simulation.

According to reference [23], the mean implantation depth *R* (unit: nm) can be calculated. The equation is shown as follows:

*R* = (40,000/*ρ*) × *E*^1.6^ (1)

where *ρ* is the density in a unit of kg/m^3^ (here we use the density of pure iron with a value of 7.86 × 10^3^ kg/m^3^); and *E* is the incident energy of positron in a unit of keV. The maximum positron mean implantation depth is about 610 nm due to the limit of the positron energy. The peaks are formed at the depth of approximately 70 nm. The damage peaks appear at almost the same depth in the samples with different fluence. Compared to the as-received samples, the *S* parameters of all post-implanted samples are larger, which means the large number of vacancy-type defects are formed after Fe^+^ irradiation.

The value of *S* parameter of the as-received samples decreases with increasing positron energy. It is because of the reduced fraction of the positrons that being trapped in the defects that will annihilate with core electrons in a manner favoring the annihilation with valence electrons [23]. Since most of irradiation-induced defects have a penetration depth of couple micron limited by the energy of implanted ions as demonstrated by the SRIM calculation, the vacancy concentration shows a depth dependent. 

The ion-implanted samples show an initial increase and then decrease in *S* parameter compared to those of the pre-irradiated samples, including one time when the *S*-*E* curves reach the peak and almost keep flattened (4–7 keV). This indicates that the defects existed in the samples and become non-homogeneously distributed with irradiation depth. The increase of *S* parameter from 1 to 4 keV can be directly attributed to the region damaged by Fe^+^ irradiation. For those with higher *E*, when implanted and thermalized positrons will not diffuse back to the surface, annihilated either in bulk or at the trapping sites, *S* value decreases with increasing energy. Similar results were reported in references [21,23,24]. The change character of *S* parameter in these three kinds of irradiated steels with increasing irradiation dose indicates that higher fluence introduces more vacancies. The *S* parameter grows with the increasing vacancy-type defects. However, the increase in irradiation dose is not obvious. It is believed that as a result of the Fe^+^ beam inducing irradiation damage tends to be saturated. The annihilated position will finally reach a value of about 0.430 J even after a low dose (1.4 × 10^14^ Fe^+^/cm^2^) Fe^+^ irradiation [24] This means that vacancy-type defects can easily be formed and even suffered from a low dose (0.2 dpa). For the samples irradiated with a fluence of 7.0 × 10^14^ Fe^+^/cm^2^, the saturation values of CNST1 and CNST2 are comparable and higher than those of CNSS3 at the same dose as shown in Figure 5d, suggesting less or smaller vacancy clusters formed in CNSS3 steel under the same experiment condition. Afterwards, a trapping model can be employed to explain these experimental results. Set the fraction of positrons annihilated in the bulk as *f_b_*, trapped and annihilated at the defects as *f_d_*, then the equations can be expressed as follows:

(2) $$f_{b}=\frac{\lambda_{b}}{\lambda_{b}+kC_{d}}$$

(3) $$f_{d}=\frac{kC_{d}}{\lambda_{d}+kC_{d}}$$

where *λ_b_* is the bulk annihilation rate; *k* is specific trapping rate for the defects and *C_d_* is defect concentration. The measured *S* parameter can be generally expressed as a linear combination of specific *S* parameter values for annihilation fractions in the bulk and in the defects, as follows:

(4) $$S_{measured}=S_{d}f_{d}+S_{b}f_{b}=\left(S_{d}-S_{b}\right)\frac{kC_{d}}{\lambda_{d}+kC_{d}}+S_{b}$$

where *S_b_* and *S_d_* are the specific *S* parameters for bulk annihilation and defect annihilation, respectively, and *f_b_* + *f_d_* = 1. Assuming *S_b_* as a constant, the value of *S_b_f_b_* will decrease (*S_d_f_d_* increase) with the increasing defect concentration, which increase with the increasing implantation ion fluence. Therefore, the measured *S* parameter from 0.5 to 7 keV increases with increasing implantation ion fluence. In the peak region of irradiation damage as shown in Figure 2, most positrons will be trapped by the defects rather than annihilated in bulk, which results in a much smaller *λ_b_* than *KC_d_*. J. Jiang [24] found that the *S_d_* was more dependent on the type of implantation ions rather than the implantation ion fluence. Since open volume of vacancy-type defects will tend to the saturation with the irradiation depth and set a top limit for the *S_d_*, it is reasonable to believe that in this experiment, Fe^+^ can easily cause the formation of vacancy-type defects.

Obtained *S-E* curves are analyzed by the VEPFIT code to calculate the depth dependence of *S* parameters. This analysis is based on the positron diffusion model using layered structures. A three-layer model is applied to describe the sample structure for the irradiated samples in Figure 5a–c. The model used divides the samples into three layers. In each layer, the *S* parameter can be fitted to obtain the agreement between the experiment and the model. Three layers identified have the boundaries of about 0–50 and 50–260 nm. The calculated depth is always deeper than the projected ranges and the simulated profiles.

For NIR layer, the *S* parameter of the positron is the same at different fluence, which are obtained from the *S*-*E* curves of the irradiated samples. The main vacancy source is located at the cascade region. However, the *S* parameter is higher for all conditions expect for CNST1 irradiated with a fluence of 3.5 × 10^14^ Fe^+^/cm^2^. This can be explained by the formation and migration of vacancy clusters. The irradiation-induced mono-vacancies migrate and aggregate to form vacancy clusters. Since vacancy diffusion is a temperature-dependent process, the diffusion of mono-vacancies in pure Fe occurs at room temperature because of its relatively low activation energy. Dimitrov et al. [25]. investigated irradiation-damage effects in a steel, which showed that the long-range migration of self-interstitial defects occurred in the range of 190–260 K temperature and the formed vacancies migrated in the range of 260–460 K temperature.

**Figure 6:**
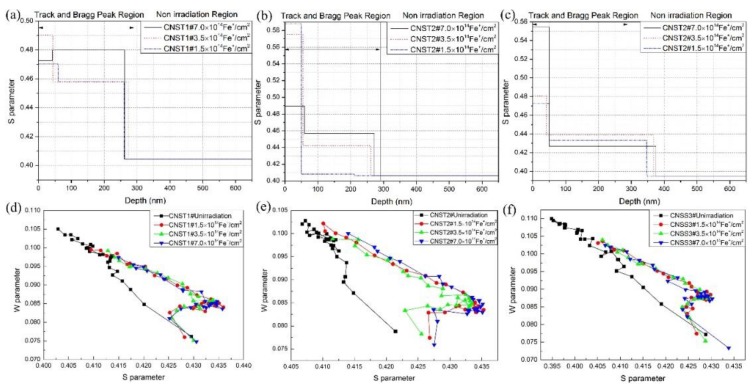
The S parameter obtained from VEPFIT vs. the positron mean implantation depth and the relationship between S-W parameter, (**a**,**d**) CNST1 steel; (**b**,**e**) CNST2 steel; (**c**,**f**) CNSS3 steel.

The defect type, which is trapped by the positrons in the materials, is revealed by plotting the *S* parameter as a function of the *W* parameter (*S*-*W* plots). If only one type of defects exists in the samples, the *S*-*W* plot can be fitted as a linear function as every kind of positron annihilation site is characterized by a typical (*S*, *W*) couple. The *S-W* plot of our experiment is shown in Figure 6, where the effects of ion dose are observed. The *S* and *W* values for pre-irradiated samples fall on a common linear locus, implying that only one type of defect exists in the as-received sample [22]. For the samples irradiated with Fe^+^, the result suggests that more than one type of defect (open-volume defect) is present in all measured irradiated alloys. As previously shown in Figure 3, point defects and small dislocation loops were discovered in irradiated samples of CNST2, which evolves from the open-volume defect promoted by Fe^+^. In addition, because carbon is considered to be very effectivity in trapping vacancies and forming vacancy-impurity complexes below 473 K, as well as the solute and impurity atoms that will combine with vacancy clusters to form vacancy-impurity complexes, it is believed that high density vacancies and vacancy clusters are formed in the samples irradiated by high energy Fe^+^.

## 4. Conclusions

The irradiation behavior of three types of HSLA steels irradiated by 400 keV Fe^+^ with different ion fluence at room temperature was investigated by TEM and PAS. The microstructure of as-received steels and irradiated samples were observed and characterized. The cross-sectional samples were prepared by FIB technique and ETE method. The main results were shown as follows:

(1) Point defects and dislocation loops were observed in the cross-sectional samples of CNST2 steel prepared by FIB but few similar defects in the sample prepared by ETE method. The bombardment of energetic focused Ga^+^ could introduce artificial defects into TEM samples that were not the actual defects induced by Fe^+^ irradiation, while ETE method provided a better way to prepare irradiated TEM samples with lesser or no damage for these materials.
(2) The distribution of vacancy clusters was heterogeneous with irradiation depth. The *S* parameter of CNST1 and CNST2 was obviously higher than that of CNSS3 at the same dose.
(3) The change character of *S* parameter in candidate steels with increasing irradiation doses was not obvious as the Fe^+^ showed a trend towards a clear saturation. The *S-W* plot showed that two types of defects were formed after ion implantation, which contained small-sized defects such as vacancies, vacancy-solute complexes, dislocation loops, and large-sized point defects.

## Figures and Tables

**Table 1:** The chemical composition of CNST1, CNST2 and CNSS3 steels (mass fraction, %).

Code	Si	Mn	P	Cu	V	Cr	Ni	Mo	Ti	C	S
CNST1	0.19	0.4	0.009	0.028	0.062	1.01	2.91	0.24	0.008	0.085	<0.005
CNST2	0.21	0.41	0.01	0.018	0.058	0.93	2.87	0.22	0.016	0.079	<0.005
CNSS3	0.26	0.58	0.007	0.092	0.059	0.99	4.14	0.46	0.01	0.1	<0.005
